# Spatial Variation in the Storages and Age-Related Dynamics of Forest Carbon Sequestration in Different Climate Zones—Evidence from Black Locust Plantations on the Loess Plateau of China

**DOI:** 10.1371/journal.pone.0121862

**Published:** 2015-03-23

**Authors:** Taijun Li, Bowen Ren, Dahui Wang, Guobin Liu

**Affiliations:** 1 College of Forestry, Northwest Agriculture and Forestry University, Yang Ling, Shaanxi, China; 2 College of veterinary medicine, Northwest Agriculture and Forestry University, Yang Ling, Shaanxi, China; 3 Institute of Water and Soil Conservation, Chinese Academy of Sciences, Yang Ling, Shaanxi, China; Tennessee State University, UNITED STATES

## Abstract

Knowledge about the long-term influences of climate change on the amount of potential carbon (C) sequestration in forest ecosystems, including age-related dynamics, remains unclear. This study used two similar age-sequences of black locust forests (*Robinia pseudoacacia L*.) in the semi-arid and semi-humid zones of China’s Loess Plateau to assess the variation in C stocks and age-related dynamics. Our results demonstrated that black locust forests of the semi-humid zone stored significantly more C than did forests in the semi-arid zone, across the chronosequence (*p* < 0.001). The C carrying capacity of the plantations was measured at 166.4 Mg C ha^−1^ (1 Mg = 10^6^ g) in the semi-humid zone, while the semi-arid zone had a capacity of only 79.4 Mg C ha^−1^. Soil organic C (SOC) increased continuously with stand age in the semi-arid zone (R^2^ = 0.84, *p* = 0.010). However, in the semi-humid zone, SOC declined sharply by 47.8% after the initial stage (5 to 10 y). The C stock in trees increased continuously with stand age in the semi-humid zone (R^2^ = 0.83, *p* = 0.011), yet in the semi-arid zone, it decreased dramatically from 43.0 Mg C ha^−1^ to 28.4 Mg C ha^−1^ during the old forest stage (38 to 56 y). The shift from being a net C sink to a net C source occurred at the initial stage in the semi-humid zone versus at the old forest stage in the semi-arid zone after reforestation. Surprisingly, with the exception of the initial and later stages (55 y), the patterns of C allocation among trees, soils, understory and litter were not statistically different between the two climate zones. Our results suggest that climate factors can alter the potential amount and age-related dynamics of forest C sequestration.

## Introduction

The global scientific community is in broad agreement that increasing carbon dioxide concentrations in the atmosphere are causing climate change with serious consequences worldwide, such as extreme climate events, rising sea levels, and disruptions in food production [1,2]. Forest ecosystems occupy more than 4.1 × 10^9^ hectares (ha) of land, and they play a central role in driving the global carbon (C) balance, due to the magnitude of their capacity for C absorption, along with their complicated spatio-temporal dynamics [3]. Thus, the forest C cycle has greatly stimulated the interests of scientists, especially in terms of the influence of climate change on the function and characteristics of forest C sequestration [4–7]. However, it remains unclear how climate change may be affecting the potential amount and age-related dynamics of forest C sequestration.

Generally, forests uptake carbon dioxide from the atmosphere by increasing plant biomass and inputting organic matter into forest soils. Photosynthesis and respiration largely control the amount of biomass accumulation in trees and litter decomposition, and those processes are affected by numerous climate factors including precipitation, temperature, and light [6,7]. Unavoidably, the trees in different climate zones differ in their ability to capture, store, and release C, which leads to variation in C sequestration in forest ecosystems, depending on the given regional climatic regime [4]. Natural climate gradients in precipitation, temperature, and other variables tend to vary systematically, and so they can enable an assessment of the potential impacts of environmental conditions on C dynamics in a given ecosystem. A number of studies have been conducted to examine the influence of climate factors on C sequestration in forests along a precipitation gradient, because this factor is an important driver of forest ecosystem functioning, as reflected in measures such as biomass production, litter mass, and soil organic C (SOC) [8–11]. Abundant rainfall is positively correlated with the production of plant biomass and the rate of litter decomposition. Moreover, such studies have demonstrated that SOC increases along a gradient of increasing precipitation [9,11]. However, these studies have not evaluated the long-term influences of different climatic conditions on the forest C cycle (such as MAP or other climate variables).

In recent years, to address climate change issues, the United Nations Framework Convention on Climate Change (UNFCCC) and Kyoto Protocol established basic principles for greenhouse emission reduction. Thus, under the background of the international negotiation on climate change, an accurate accounting of the C storage has become a critical challenge [12,13]. The space-time substitution method and forest inventory date (FID) are widely applied to the study of the C sequestration dynamics and storage in forest ecosystems at a large scale. Previous studies have evaluated C stocks in many forest types and their dynamics across forest chronosequences [14–17]. These studies have revealed the characteristics of forest C sequestration on storage, accumulation rate, allocation pattern, and the age-related dynamics, during stand development. Current studies hypothesize that these characteristics are consistent across space for a given forest type. However, the majority of studies were conducted in a single climate zone, so it is not known whether the findings are applicable to different climate zones for any given forest ecosystem. To support the development of environmental research and policies about C assessment and management at national and global scale, it is also essential to clearly understand the long-term influence of climate zone on forest C sequestration [18].

In this study, we selected black locust forests (*Robinia pseudoacacia L*.) on the Chinese Loess Plateau to explore the influence of different climate zones on variation in the potential and forest age-related dynamics of C sequestration. Black locust trees are planted widely on the Loess Plateau and the northern regions of China as a means to address the intertwined environmental problems of climate change, drought and erosion, because this species has an extensive root system and a considerable capacity for nitrogen fixation [19]. Thus, this forest type also has a remarkable ability to sequester and store C in resource-constrained environments [20]. The objectives of this work were to test the following predictions: (1) if the C carrying capacity of black locust forest is significantly greater in the Loess Plateau’s semi-humid zone than in semi-arid zone; (2) if the age-related dynamics and allocation patterns of C sequestration in black locust forest vary between different climate zones.

## Materials and Methods

### Ethics Statement

The study obtained ethical approval from the Academic Committee of the Institute of Soil and Water Conservation, Chinese Academy of Sciences. All the research was approved and supervised by local authorities of state-owned forestry bureaus. Our study was conducted neither in privately-owned forest, nor in biosphere nature reserves. Additionally, all the samplings did not involve protected or endangered animals and plants.

### Site description and experimental design

We implemented this study in Ansai and Yongshou counties, which are located in the central and southern region of the Loess Plateau in Shaanxi Province, China (Ansai: 108° 5′–109° 26′ E, 36° 30′–37° 19′ N; Yongshou: 107° 56′–108° 21′ E, 34° 29′–34° 59′ N). Ansai county has a temperate, semi-arid climate with a MAP of 505.3 mm (varying from 700 mm in wet years to 300 mm in dry years), most of which is highly erosive and occurs intensively from July to September. Its mean annual temperature (MAT) ranges from 7.7 to 10.6°C, with a minimum temperature of −24°C in January and maximum of 37°C in July. The average frost-free period is 157 days, and the total yearly sunshine is 2415 h. The general property of the loess soil profile in the study area consists of 65% sand, 24% silt, and 11% clay. The second county, Yongshou, occurs on part of the gully and rolling loess region of Northern Weinan. It has a typical warm temperate climate, with a MAP of 606.1 cm (varying from 857.3 mm in wet years to 298.9 mm in dry years). Approximately 52% of rainfall also occurs intensively from July to September as torrential rain. The MAT is 10.8°C with a minimum of −18°C in January and a maximum of 38.9°C in July. Its average frost-free period is 210 days and total yearly sunshine is 2166 h.

Black locust forest is one of the dominant planted forest types on the Loess Plateau and widely distributed at the different climate zones of Loess Plateau. In our study, we carefully chose the sampling sites to ensure similar soil conditions and human management characteristics, with the aim of testing the influence of climate zone on C sequestration in the plantations. Based on governmental records of reforestation, and oral testimonies from local elders, similar age-sequences of black locust forests at the two sites were compared to determine the C stock in forest ecosystems at different stages of stand development. The stand ages were 5, 9, 20, 30, 38, and 56 y in Ansai county (semi-arid zone, hereafter “drier zone”), and 5, 10, 20, 30, 44, and 55 y in Yongshou county (semi-humid zone, hereafter “wetter zone”). To meet the assumptions of the chronosequence approach, the abiotic and biotic factors in these age-sequences were controlled as much as possible. Soil properties, stand density, stand management and water regime were similar among the sampling sites, as were the aspect, slope position and gradient (Table 1).

**Table 1:** Details of the samplings in two sites.

Sites	Forest age (y)	Altitude (m)	Gradient (°)	Aspect	Slope position	Dominant undergrowth plants
Ansai county	5	1260–1287	22–30	SW^♀^	Upper	*Rosaceae saxatilis*
	9	1161–1228	27–33	SW	Upper	*Roegneria kamoji*
	20	1236–1259	17–25	SSW^♂^	Middle	*Viola phitippica*
	30	1208–1244	22–25	SSW	Upper	*Artemisia annua*
	38	1185–1227	18–22	SW	Upper	*Urena lobata*
	56	1170–1175	21–24	SSW	Middle	*Setaria viridis*
Yongshou county	5	1230–1257	15–21	SSW	Upper	*Lespedeza bicolor*
	10	1185–1240	20–23	SW	Middle	*Arex duriuscula*
	20	1208–1256	17–22	SSW	Upper	*Pyrularia edulis*
	30	1140–1195	14–19	SW	Middle	*Artemisia scoparia*
	44	1213–1257	21–24	SSW	Upper	*Potentilla discolor*
	55	1190–1234	18–23	SSW	Upper	*Setaria viridis*

♀SW: southward

♂SSW: semi-southward

### Measurement of plant biomass

Sampling took place from July to September 2011 in the drier zone and August 2012 in the wetter zone. In the field, sample plots (20 m × 30 m in drier zone and 20 m ×50 m in wetter zone) were established to estimate the C storage with three duplicate plots and the diameter at breast height (DBH, measured at 1.3 m above ground level) of each tree was measured and recorded in each point. Li *et al*. [21] have developed a series of allometric models to estimate the black locust trees biomass on Loess Plateau. Because of the difficulty in accurately measuring the tree heights of dense stands in loess hilly area, in their study the single independent variable of DBH was involved into the ln-transformation models as follows:

Ln(M)=a+bLn(DBH) (1)

where *M* and *DBH* represent the tree component biomass (kg) and the tree diameter at breast height cm), respectively. The value of a and b are the allometric scaling coefficients of intercept and slope, and was estimated by employing Least squares linear regression. According to the strong logarithmic linear correlation between tree biomass and DBH, the biomass of each tree component (including leaf, branch, stem without bark, bark, and root) was calculated after measuring the DBH. The total biomass of the trees was calculated by summing the biomass of individual trees. A total of 36 plots from six age stands in two sites were assessed.

A harvesting approach was applied to calculate the secondary biomass C (SBC, includes shrub, herbage, and leaf litter) of black locust forest. Shrub biomass was measured in 2 m × 2 m subplots along a diagonal of sampling quadrate, while herbage and some lianas were measured in 1 m × 1 m subplots along the other diagonal. There were 3 replicates in each shrub and herbage subplot. The fresh weight of above-ground and below-ground biomass for each dominant plant species was harvested. Each sub-sample was taken to the laboratory and dried in an oven at 65°C for 72 h to estimate its dry matter content. In addition, sub-samples of ~0.5 kg of every sample were taken to the laboratory in order to determine the carbon fraction they contained.

### Soil sampling and calculation of soil organic carbon

In each plot, one soil profile was established and five core-samples were taken down to a depth of 100 cm in the mineral soil. A soil corer of 5-cm diameter was used to collect soil samples at 5 locations along an “S” curve from the bottom to top of the sloping surface. The cores were divided into 5 layers: 0–10, 10–20, 20–30, 30–50, and 50–100 cm. All five samples from the same depth layer in each plot were combined into a single sample to measure the soil C content at that depth. Soil samples were dried at 55°C for 72 h, and subsequently ground and passed through a 0.2 mm sieve. C content in mineral soil was determined in samples dried at 70°C for 48 h, using a C/N elemental analyzer [22] in an auto analyzer. The soil profile samples were used to measure bulk density, by calculating the mass of 100 cm^3^ of dried soil after removing any stones, gravel, and fine roots.

The total amount of C stored in the soil was quantified based on the C content of soil layers, their bulk density and sampling depth, as follows:

$$SOC=\sum_{i=1}^{n}depth_{i}\text{· }CC_{i}\text{· }BD_{i}$$ (2)

where *SOC* is the total soil organic C stock (Mg C/ha); *CC*_*i*_ is the C concentration in soil layer *i* (g C/kg); *BD*_*i*_ is the bulk density in layer *i* (g/cm^3^); *depth*_*i*_ is the sampling depth in layer *i* (cm); and *i* is each of the five soil sampling layers.

### Statistical analysis

Means and standard errors of the biomass C and SOC were calculated from replicates (3 for biomass C measurements, and 5 for SOC). Regression analyses were conducted to fit the relationship between C stock and forest age. The coefficient of determination (*R* ^*2*^) and the level of probability (*P*) were used to determine the goodness of fit of the curves. The least significant difference (LSD) test was performed to test for differences in C stocks in stands of similar ages between the two climate zones. The level of significance *P* ≤ 0.05 was used in the analysis of variance (ANOVA). All statistical analyses were performed using SPSS 13.0 for windows (SPSS Inc., Chicago, Illinois, USA, 2004).

## Results

### Carbon sequestration in black locust forests

**Fig 1:**
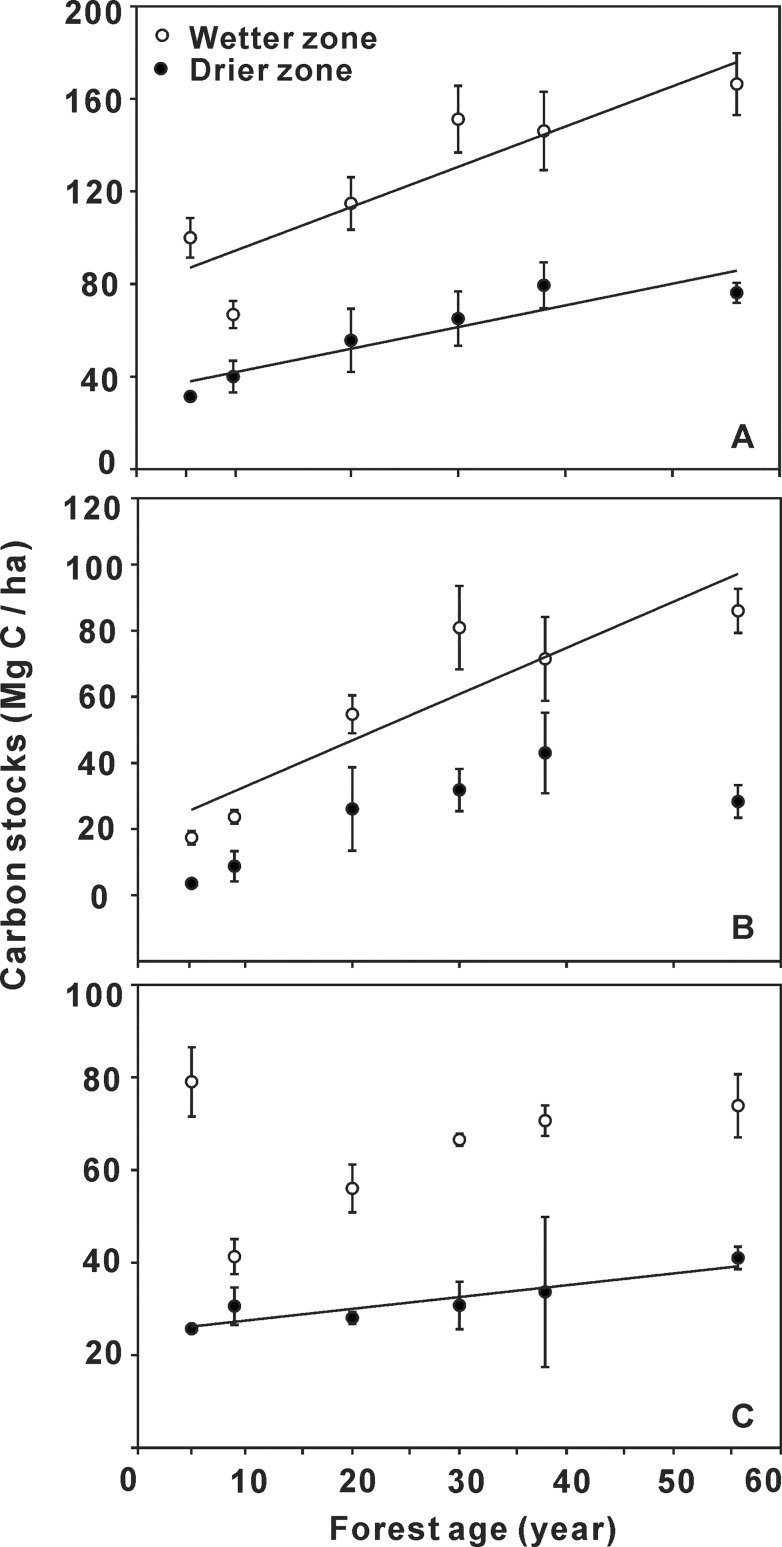
Carbon stocks in ecosystem (A), tree biomass (B), and forest soil (C) of black locust plantations across the chronosequence in drier zone and wetter zone of the Loess Plateau. Linear regression relationship between C stock and forest age was: (A) ecosystem, y = 78.39 + 1.74 x (R^2^ = 0.79, *P* = 0.017) in wetter zone and y = 33.28 + 0.94 x (R^2^ = 0.85, *P* = 0.008) in drier zone, respectively; (B) tree biomass, y = 18.86 + 1.40 x (R^2^ = 0.83, *P* = 0.011) in wetter zone; (C) forest soil, y = 24.90 + 0.26 x (R^2^ = 0.84, *P* = 0.010) in drier zone. Values are represented by mean ± standard deviation and the duplicates are 3 in drier zone and 5 in wetter zone, respectively. Significance level was determined at P ≤ 0.05.

There was a significant linear relationship between black locust forest C stocks and stand age in the drier zone (R^2^ = 0.79, *P* = 0.017) and in the wetter zone (R^2^ = 0.85, *P* = 0.008) (Fig. 1A). The C carrying capacity of plantations were 79.4 Mg C ha^−1^ (1 Mg = 106 g, 38 y, S1 Table) and 166.4 Mg C ha^−1^ (55 y, S2 Table), with the rates of C accumulation were 0.9 Mg C ha^−1^ per year and 1.3 Mg C ha^−1^ per year in the respective sites (Fig. 1A).

The slope of the regression line (R^2^ = 0.83, *P* = 0.011) for C stock in tree biomass against forest age, in the wetter zone, indicated a substantial increase from 17.4 Mg C ha^−1^ (5 y) to 86.0 Mg C ha^−1^ (56 y) (Fig. 1B, S2 Table). However, the C stock in trees increased from 3.6 Mg C ha^−1^ at 5 y to 43.0 Mg C ha^−1^ at 38 y in the drier zone, followed by a significant decrease to 28.4 Mg C ha^−1^ at 56 y (*P* < 0.01) (Fig. 1B, S1 Table).

Soil organic C decreased dramatically by about 47% (from 79.0 Mg C ha^−1^ to 41.3 Mg C ha^−1^) in the initial stage (5 y to 10 y) of reforestation in the wetter zone (Fig. 1C, S2 Table), resulting in a dramatically decrease from 100.0 Mg C ha^−1^ to 66.8 Mg C ha^−1^ in total ecosystem C stock (Fig. 1A, S2 Table). It suggested that black locust forests there shifted from being a net C sink to a C source in the initial stage of reforestation in the wetter zone. At later stand ages, SOC stocks increased gradually to 73.9 Mg C ha^−1^, and this value was still smaller than that in the 5-y-old stand (S2 Table). However, there was a significant linear relationship between SOC stock and forest age in the drier zone (R^2^ = 0.84, *P* = 0.010), increasing steadily from 25.7 Mg C ha^−1^ to 41.0 Mg C ha^−1^ (S1 Table).

**Fig 2:**
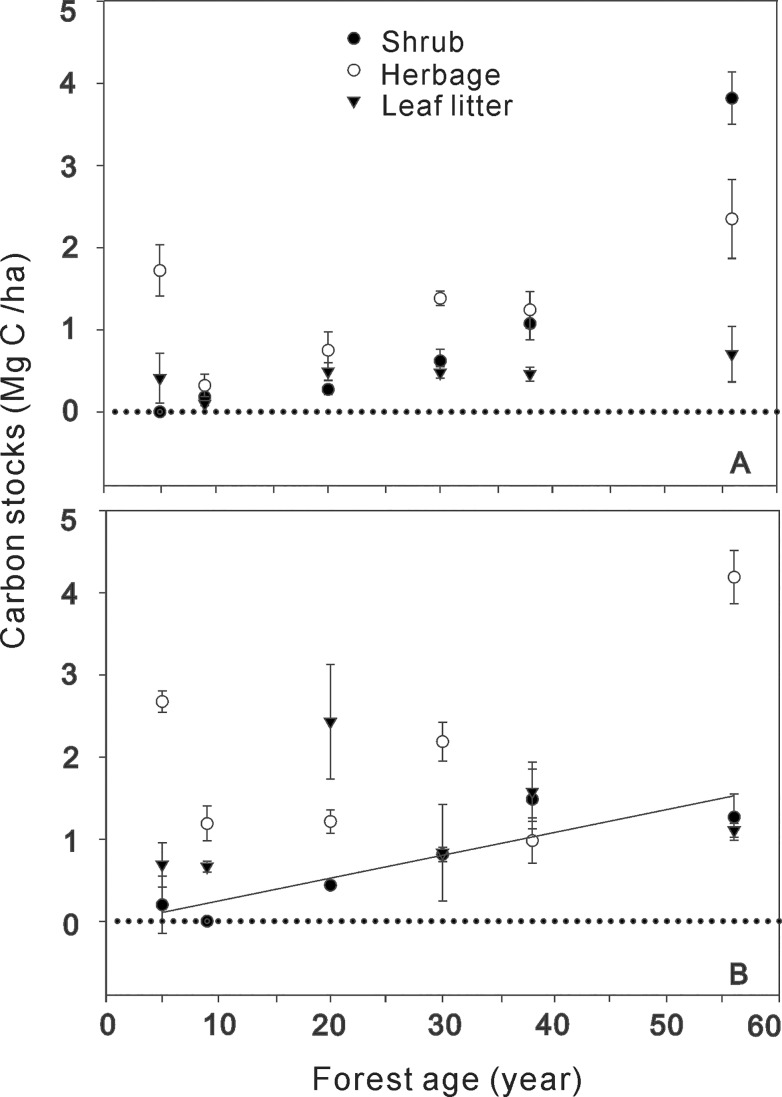
Secondary biomass C (SBC) of black locust plantations in drier (A) and wetter zone (B) across the chronosequence. Shrub C stock against forest age in wetter zone (B) performed significant linear regression relationship as follow: y = −0.033 + 0.028 x (R^2^ = 0.80, *P* = 0.01). The values of C stock lie on the level of zero represent that these components was not found in the plantations; the bars represent the standard deviation.

Secondary biomass C (SBC) was apparently independent of stand age (Fig. 2A and B). However, the C stock in shrubs of the wetter zone increased linearly and significantly with forest age (Fig. 2B). The largest SBC occurred at the old forest stage with 6.9 Mg C ha^−1^ (56 y) in the drier zone, including 3.8 Mg C ha^−1^ in shrubs, 2.4 Mg C ha^−1^ in herbages, and 0.7 Mg C ha^−1^ in leaf litters (S1 Table). Likewise, SBC in the 55-y-old stand was also the largest (6.6 Mg C ha^−1^) in the wetter zone, which consisted of 1.3 Mg C ha^−1^ in shrubs, 4.2 Mg C ha^−1^ in herbages, and 1.1 Mg C ha^−1^ in leaf litters (S2 Table). Although the stand age-related dynamics of SBC were not consistent, the findings still suggested that a considerable addition of SBC occurred at later stages of reforestation. However, C stocks in shrubs, herbages and leaf litters were all at a low level in both climate zones, across the chronosequence (Fig. 2, S1 and S2 Table).

### Spatial variation of carbon stocks in different stand ages

**Fig 3:**
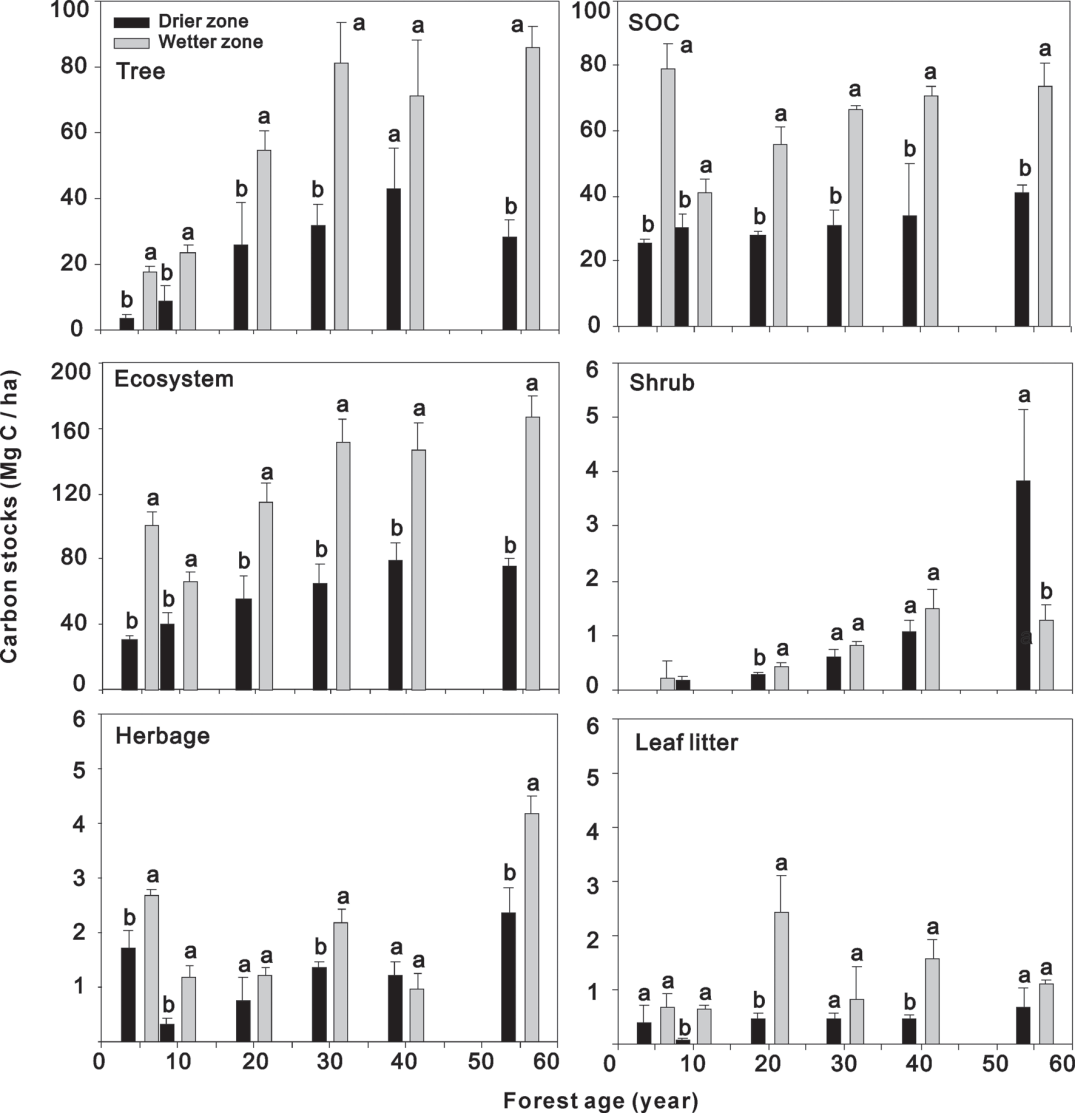
Spatial variety of component C stocks between drier region and wetter region across the chronosequence. ANOVA test was conducted under *P* < 0.05, the bars represent standard deviation and the letters represent the significance of difference, no shrub was found at 5-y-old stand in drier zone and at 10-y-old stand in wetter zone, respectively.

Carbon stocks in the wetter zone were significantly larger than those in the drier zone (including ecosystem C, tree biomass C, and SOC) (Fig. 3). The maximum capacity of ecosystem C sequestration was observed in the 38-y-old stand (79.4 Mg C ha^−1^) of the drier zone, where the largest C stock in trees was also found (43.0 Mg C ha^−1^) (Fig. 3, S1 Table). In the wetter zone, the maximum capacity of ecosystem C sequestration was found in the 55-y-old stand (166.4 Mg C ha^−1^), and the largest SOC stock was found at the initial stage of reforestation (5 y, 79.0 Mg C ha^−1^) (S2 Table). However, SOC decreased dramatically, and then gradually returned to close to the initial amount (55 y, 73.9 Mg C ha^−1^). Notably, SBC stocks may not only be independent of forest age (Fig. 2), but also independent of their regions of distribution (Fig. 3).

### Patterns of carbon allocation in different climate zones

The contribution of tree biomass C to ecosystem C stocks increased continuously in black locust plantations of both sites, from 17% (5 y) to 53% (30 y) in the wetter zone and from 11% (5 y) to 55% (38 y) in the drier zone, respectively (Table 2). Forest soils contributed the majority of C at the initial stage of reforestation (82% in the drier zone vs. 79% in the wetter zone) (Table 2). However, their contribution decreased to approximately 50% over time, due to the rapid growth of trees. In particular, the proportion of tree biomass C decreased notably from 38 to 56 y in the drier zone (Table 2). SBC played a negligible role in C sequestration in plantations, and the biggest contribution (7%, 5 y) was found at the initial stage of reforestation in the drier site (Table 2).

**Table 2:** Dynamics of C allocation of plantations in the drier zone and the wetter zone.

	Proportion /Tree			Proportion /Soil			Proportion /SBC^⊕^		
Age (y)	DZ^♀^	WZ^♂^	P	DZ	WZ	P	DZ	WZ	P
5	0.11(0.02)	0.17(0.02)	0.02	0.82(0.02)	0.79(0.02)	0.12	0.07(0.00)	0.04(0.00)	0
9/10§	0.26(0.04)	0.35(0.00)	0.01	0.72(0.04)	0.62(0.00)	0.01	0.02(0.00)	0.03(0.00)	0.05
20	0.45(0.10)	0.48(0.00)	0.68	0.52(0.09)	0.49(0.01)	0.58	0.03(0.01)	0.04(0.01)	0.56
30	0.49(0.02)	0.53(0.04)	0.10	0.48(0.01)	0.44(0.04)	0.12	0.03(0.01)	0.03(0.01)	0.05
38/44	0.55(0.16)	0.48(0.06)	0.56	0.42(0.16)	0.49(0.06)	0.54	0.04(0.01)	0.03(0.01)	0.1
56/55	0.39(0.05)	0.52(0.01)	0.02	0.56(0.04)	0.44(0.01)	0.01	0.05(0.02)	0.04(0.00)	0.21

The proportion was the ratio of the component’ C stock to the total ecosystem C stock, denoted by the values with standard errors between parentheses; *P* values represent the results of ANOVA on proportions between two sites at the similar forest ages, all the *P* values were conducted under 0.05 level.

§9/10, represents 9-y-old in the drier zone and 10-y-old in the wetter zone

♀DZ: drier zone

♂ WZ: wetter zone

⊕SBC: secondary biomass carbon, was constituted of shrubs, herbages, and leaf litters of plantation

Significant differences in the C allocation to trees and forest soil were observed in both the initial and old forest stages (approximately 40 to 55 y) between the two sites (*P* < 0.05) (Table 2). However, this effect was not observed at other stand stages, suggesting no influence of climate zone on C allocation from ages of 20 to 40 y (*P* > 0.05) (Table 2). The proportion of SBC to ecosystem C stocks was apparently independent of both age and distribution zone.

**Fig 4:**
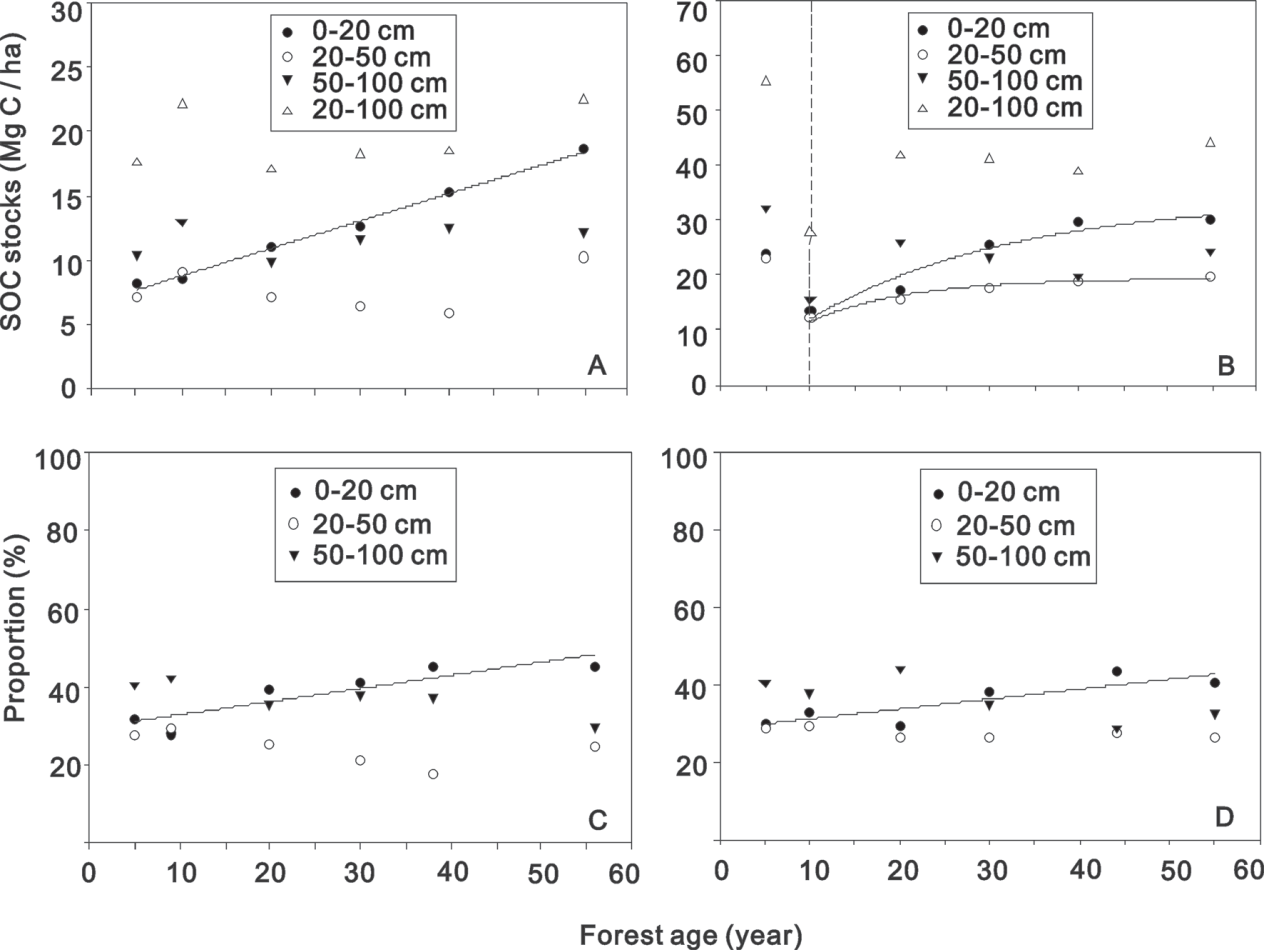
Soil organic C stocks (A, B) and the proportions of each layer’ SOC stock to total SOC (C, D) in different soil depths across the chronosequence in two climate zones. (A): drier zone, y = 6.68 + 0.21 x (R^2^ = 0.99, *P* < 0.001; 0–20 cm); (B): wetter zone, y_1_ = 33.84 * (1—exp (−0.04 x)) (R^2^ = 0.94, *P* = 0.005, 0–20 cm), y_2_ = 19.42 * (1—exp (−0.09 x)) (R^2^ = 0.98, *P* = 0.002, 20–50 cm), x∈[10, 55], the dotted line separates two stages of C stock change during the stand development; (C): drier zone, y = 0.297 + 0.003 x (R^2^ = 0.80, *P* = 0.016, 0–20 cm); (D): wetter zone, y = 0.285+ 0.003 x (R^2^ = 0.76, *P* = 0.024, 0–20 cm).

The SOC stocks in surface soils (depth 0–20 cm) increased linearly with forest age in the drier zone (Fig. 4A), but such patterns were not observed at other soil depths (Fig. 4A, S3 Table). In the wetter zone, the SOC stock at each depth decreased markedly from 5 to 10 y, while the total SOC stock decreased from 79.0 Mg C ha^−1^ to 41.3 Mg C ha^−1^ (S2 Table). However, the SOC stock in the 0–20 cm and 20–50 cm layers increased exponentially to 29.9 Mg C ha^−1^ and 19.7 Mg C ha^−1^, respectively, from 10 y to 55 y (Fig. 4B, S4 Table). The proportion of surface SOC (0–20 cm) to total SOC increased continuously from 31.7 to 45.3% in the dried zone (Fig. 4C), and from 30.2 to 40.5% in the wetter zone (Fig. 4D).

## Discussion

### Spatial variation in the carbon storages between climate zones

Generally, abundant rainfall is related to higher net primary production in terrestrial ecosystems [7,23]. Higher precipitation always leads to faster growth of trees and more accumulation of organic matter into forest soils, resulting in larger C stores in forests [24]. Thus, we predicted that the stocks and C carrying capacity of forest C sequestration would be larger in the wetter zone. Indeed, our results demonstrated that C stocks in black locust forest on the Loess Plateau were significantly larger in the wetter zone (Fig. 3). Furthermore, the C carrying capacity of plantation was approximately double in the wetter zone than that of the drier zone (166.4 Mg C ha^−1^ and 79.4 Mg C ha^−1^, Fig. 3).

Although we still lack sufficient information about the long-term impacts of precipitation variation on the accumulation of forest C, previous studies of precipitation gradients contain valuable data that relates to our predictions. Thus, these studies seem to support the notion that increased precipitation facilitates biomass production in forests [23,25,26]. Our results agree with those findings—the tree biomass C stocks were significantly larger in the wetter zone that we studied. In addition, much evidence shows that total ecosystem C increases with increasing MAP [25,27]. We found that total ecosystem C stocks were also noticeably larger in the wetter zone of the Loess Plateau (Fig. 3).

Spatial variation in precipitation always results in a different amount of litter input and fine root turnover. Large differences in SOC stocks were also found between the two climate zones of our study, where the ratio of SOC in the wetter zone to the drier zone ranged from 1.3 to 3.1 at different forest ages (Fig. 3). Meier *et al* (2010) reported that SOC decreased by about 25% in European beech (*Fagus sylvatica L*.) stands when annual precipitation ranged from > 900 mm to < 600 mm, along a natural moisture gradient [28]. Apparently, the intensity of changes in SOC stocks from wetter to drier conditions was much larger in our study than was reported by Mei *et al*. [28]. A similarly intense change was found in West Africa, where SOC stocks in the upper 2 m of soil ranged from 20 to over 120 Mg C ha^−1^ [11]. However, this result was derived from a long-distance latitudinal transect, combined with the effects of climate and vegetation types [11]. The response of ecosystem C processes to variation in precipitation is highly complex [24,29,30]. In another study, SOC remained relatively constant along the precipitation gradient [10]. Carbon accumulation in forest soil was controlled by various factors, including climate, vegetation type, and forest management practices. In water-limited regions, precipitation may be the most important factor driving the great variation in SOC content.

A number of large-scale studies that have estimated forest C stocks potentially assumed that C storage in a forest ecosystem in a given region is constant across space, especially those based on forest inventory data (FID) [31,32]. However, C storage in black locust plantations on the Loess Plateau showed a contrary pattern. We know that precipitation plays a crucial role in forest C cycling, influencing both the stocks and potential of C sequestration. Our study suggests that measures of forest C sequestration based on a single climate zone should be extrapolated to large-scale C storage estimation only with great caution. Incorporating the variability of forest C sequestration into forest C cycle also apparently improves the accuracy of regional C stock evaluation and forest productivity prediction.

### Climate effects on age-related carbon dynamics

Reforestation is widely accepted as an effective measure to enlarge terrestrial C sinks [18,33]. The accumulation of forest C is related to stand age, resulting from the rapid growth of trees and the augmentation of SOC. In our study, C storage in planted forests increased significantly with stand age after reforestation, in both drier and wetter zones of the Loess Plateau (*P* < 0.01, Fig. 1A). From sapling stands to old forest, the amount of ecosystem C increased by 142% and 66% in the drier and wetter zone, respectively. These were similar to the findings of a number of other studies that have used the chronosequence approach [14,16,17,34–36].

However, we found that the age-related C dynamics of SOC stocks and tree biomass C varied between the drier and wetter zones of the Plateau (Fig. 1B and C). The shift from being a net C sink to a net C source appeared to occur at the sapling stage in the wetter zone, but not until the old forest stage in the drier zone. In the wetter zone, SOC accounted for the majority of total ecosystem C in sapling stands, and its fluctuation played an important role in total ecosystem C dynamics. Lu *et al*. (2013) reported that SOC decreased in the first few years after black locust trees were planted on the Loess Plateau, where the magnitude of decrease was large in wetter sites (MAP ~600 mm) and small in drier sites (MAP from ~450–500 mm) [37]. Consistently, planting trees has been shown to disturb soil texture and enhance the amount of soil microbiological activity, resulting in the release of some soil C stores to the atmosphere [8]. However, an increasing body of evidence on SOC change suggests that the influence of reforestation on SOC may depend on MAP [38]. The results observed in former grasslands planted with *Eucalyptus* have demonstrated that drier sites gained SOC and wetter sites lost SOC [8]. In addition, SOC tends to be lost above a MAP threshold of ~600 mm [39]. Our results support these findings. In our study, SOC in planted forest increased continuously in the drier zone (from 25.7 Mg C ha^−1^ to 41.0 Mg C ha^−1^), whereas SOC decreased slightly in the wetter zone (*P* > 0.05) (Fig. 3, S3 and S4 Table).

The relationship between tree biomass C and forest stand age also differed between the two climate zones. Planted forest continually accumulated C in tree biomass, in a linear relationship with stand age in the wetter zone (Fig. 1B). However, tree biomass C dramatically decreased in the drier zone, although forest soils accumulated C consistently (Fig. 3). Thus, total ecosystem C decreased slightly, by approximately 5% (Fig. 3), suggesting that planted forest became a potential C source in the later stage. Old forests always release C to the atmosphere when a mass of trees are damaged [40]. In contrast, previous studies have reported that old forest can accumulate C in forest soils [41,42]. Perhaps both the results of previous studies and our investigation of black locust plantations on the Loess Plateau are supported, if we infer that whether old forests are able to consistently accumulate SOC depends on the amount of precipitation (Fig. 1C).

The pattern of ecosystem C allocation to plant and soil changed with forest age, likely resulting from the counterbalance in the relative contributions of plant biomass C and SOC (Table 2). Because of the rapid growth of trees, the proportion of tree biomass C to total ecosystem C increased steadily in both climate zones (Table 2). Furthermore, those proportions in the two sites were significantly different between the initial stage (5 y and 10 y) and the later stage (55 y). As mentioned above, the age-related dynamics of tree biomass C and SOC resulted in a surprising allocation of C to plants and soils (Fig. 1). However, our results still demonstrated that the pattern of C allocation remained relatively constant between stand ages of 20 to 40 y, among trees, soil, and SBC (Table 2). Our results seem to indicate that the climate zone does not alter the pattern of C allocation through stand development from the middle to mature stages, at least at the geographic scale and level of climate difference that we studied. This finding will support the development of allocation rules to accurately predict C pools and fluxes in planted forests.

In addition, our study will also contribute to a better understanding of the potential influence of climate change on forest C cycling. For example, in our study the maximum capacity of forest C stocks during stand development significantly decreased with a decrease in MAP. It appears to indicate that MAP change in semi-arid regions alters the potential capacity of forest ecosystems in sequestrating carbon dioxide from atmosphere. Thus, we hypothesize this phenomenon would also affect the dynamics of regional C balance. However, although black locust plantations showed an interesting change in the C storages and allocation due to MAP change, it still lacks sufficient date about whether natural forests are consistent with our results. Further research in the long term influence of MAP changes is required to further test our hypothesis. On the other hand, temperature always plays a key role in forest productivity and litterfall decomposition and is related to the C accumulation in plant biomass and soil. However, in arid and semi-arid regions, precipitation is the dominant factor for C balance [24]. Furthermore, a long-term study in the boreal forests of western Canada has also demonstrated that drought-induced water stress due to climate change results in an observed reduction in the biomass C sink [7]. Additionally, the western Canada’ boreal forests may become net C sources when induced droughts continue to intensify [7]. Our results were consistent with a previous study, highlighting that in the drier zone biomass C dramatically decreased at the old forest stage and forest released C into atmosphere (Fig. 1).

## Conclusions

Our study demonstrated that the C storage in black locust forests was significantly larger in the wetter zone than in the drier zone (*P* < 0.001). The carbon carrying capacity of plantation was also approximately double in the wetter zone (166.4 Mg C ha^−1^ versus 79.4 Mg C ha^−1^). The mean rates of C accumulation in the respective zones were 1.3 Mg C ha^−1^ per year and 0.9 Mg C ha^−1^ per year. Soil organic C (SOC) stocks increased continuously in the drier zone. However, the SOC stock sharply decreased at the initial stage of reforestation in the wetter zone. It suggests that whether old forest is able to accumulate C in soils depends on the climate zone. Tree C stocks increased linearly with stand age in the wetter zone. However, in the drier zone, they decreased markedly at the old forest stage. It should be noted that black locust forest shifts from being a net C sink to a net C source after reforestation in both zones, although this occurs at different stages depending on the climate (at the initial stage in humid conditions and the old forest stage in dry conditions). However, with the exception of the initial and later stages, the patterns of C allocation to trees, soils and SBC were independent with climate zones. Moreover, the proportion of surface SOC (at 0–20 cm depth) to total SOC increased steadily with increasing stand age in both climate zones. Our results suggest that regional climate factors may play a complicated role in the potential amount and age-related dynamics of forest C sequestration. Notably, the C sequestration characteristics of a forest ecosystem located in a given climate zone should be used only cautiously for extrapolated estimates of the storage and potential of regional C sequestration.

## Supporting Information

S1 Table Carbon pools of black locust forests in semi-arid zone (Ansai county). Carbon stocks are represented as mean value (standard deviation) (Mg C ha^−1^).
(DOC)

S2 Table Carbon pools of black locust forests in semi-humid zone (Yongshou county). Carbon stocks are represented as mean value (standard deviation) (Mg C ha^−1^).
(DOC)

S3 Table Soil organic carbon stocks of black locust forests in semi-arid zone (Ansai county). (DOC)

S4 Table Soil organic carbon stocks of black locust forests in semi-humid zone (Yongshou county). (DOC)
