# Supplementary material for: Spatial Variation in the Storages and Age-Related Dynamics of Forest Carbon Sequestration in Different Climate Zones—Evidence from Black Locust Plantations on the Loess Plateau of China
Source: PLoS One. 2015 Mar 23;10(3):e0121862. doi: 10.1371/journal.pone.0121862 (PMC4370400; doi:10.1371/journal.pone.0121862)
Supplement: S1 Table — Carbon stocks are represented as mean value (standard deviation) (Mg C ha−1). (DOC) [file pone.0121862.s001.doc]

S1 Table. Carbon pools of black locust forests in semi-arid zone (Ansai county). Carbon stocks are represented as mean value (standard deviation) (Mg C ha^-1^).

| Components | Forest age (year) | | | | | |
| --- | --- | --- | --- | --- | --- | --- |
|  | 5 | 9 | 20 | 30 | 38 | 56 |
| Trees | 3.59(0.90) | 8.80(4.58) | 26.09(12.58) | 31.83(6.37) | 43.00(12.18) | 28.35(4.91) |
| Shrubs | 0 | 0.18(0.05) | 0.27(0.06) | 0.62(0.14) | 1.08(0.20) | 3.82(1.32) |
| Herbages | 1.72(0.31) | 0.32(0.14) | 0.75(0.42) | 1.38(0.09) | 1.24(0.23) | 2.35(0.48) |
| Litters | 0.41(0.30) | 0.10(0.02) | 0.49(0.11) | 0.48(0.07) | 0.46(0.08) | 0.70(0.34) |
| SOC | 25.69(0.85) | 30.58(4.04) | 28.03(1.34) | 30.76(5.13) | 33.66(16.19) | 41.00(2.42) |
| Ecosystem | 31.42(1.82) | 39.98(6.88) | 55.63(13.64) | 65.07(11.76) | 79.44(9.95) | 76.21(4.38) |

Note: SOC is soil organic carbon.
