# Supplementary material for: Spatial Variation in the Storages and Age-Related Dynamics of Forest Carbon Sequestration in Different Climate Zones—Evidence from Black Locust Plantations on the Loess Plateau of China
Source: PLoS One. 2015 Mar 23;10(3):e0121862. doi: 10.1371/journal.pone.0121862 (PMC4370400; doi:10.1371/journal.pone.0121862)
Supplement: S2 Table — Carbon stocks are represented as mean value (standard deviation) (Mg C ha−1). (DOC) [file pone.0121862.s002.doc]

S2 Table. Carbon pools of black locust forests in semi-humid zone (Yongshou county). Carbon stocks are represented as mean value (standard deviation) (Mg C ha^-1^).

| Components | Forest age (year) | | | | | |
| --- | --- | --- | --- | --- | --- | --- |
|  | 5 | 10 | 20 | 30 | 44 | 55 |
| Trees | 17.41(2.09) | 23.65(2.08) | 54.73(5.72) | 80.84(12.61) | 71.43(16.68) | 85.95(6.63) |
| Shrubs | 0.20(0.35) | 0.00 | 0.44(0.05) | 0.81(0.09) | 1.49(0.36) | 1.27(0.28) |
| Herbages | 2.67(0.13) | 1.19(0.21) | 1.21(0.14) | 2.19(0.24) | 0.98(0.27) | 4.19(0.32) |
| Litters | 0.69(0.27) | 0.66(0.07) | 2.43(0.70) | 0.83(0.59) | 1.58(0.36) | 1.11(0.09) |
| SOC | 79.03(7.49) | 41.28(3.79) | 56.01(5.18) | 66.54(1.31) | 70.63(3.31) | 73.87(6.83) |
| Ecosystem | 100.01(8.55) | 66.79(5.87) | 114.82(11.03) | 151.21(14.43) | 146.11(16.93) | 166.37(13.41) |

Note: SOC is soil organic carbon.
