# Supplementary material for: Spatial Variation in the Storages and Age-Related Dynamics of Forest Carbon Sequestration in Different Climate Zones—Evidence from Black Locust Plantations on the Loess Plateau of China
Source: PLoS One. 2015 Mar 23;10(3):e0121862. doi: 10.1371/journal.pone.0121862 (PMC4370400; doi:10.1371/journal.pone.0121862)
Supplement: S3 Table — (DOC) [file pone.0121862.s003.doc]

S3 Table. Soil organic carbon stocks of black locust forests in semi-arid zone (Ansai county).

| Forest ages (year) | Carbon stocks of each soil layer (Mg C ha^-1^) | | | | |
| --- | --- | --- | --- | --- | --- |
|  | 0－10 cm | 10－20 cm | 20－30 cm | 30－50 cm | 50－100 cm |
| 5 | 4.79 | 3.35 | 2.59 | 4.55 | 10.43 |
| 9 | 4.90 | 3.64 | 3.10 | 5.96 | 12.97 |
| 20 | 7.03 | 3.98 | 2.79 | 4.30 | 9.94 |
| 30 | 8.37 | 4.24 | 2.98 | 3.49 | 11.67 |
| 38 | 10.67 | 4.53 | 3.27 | 2.66 | 12.53 |
| 56 | 12.07 | 6.51 | 4.53 | 5.71 | 12.17 |
