# Supplementary material for: Spatial Variation in the Storages and Age-Related Dynamics of Forest Carbon Sequestration in Different Climate Zones—Evidence from Black Locust Plantations on the Loess Plateau of China
Source: PLoS One. 2015 Mar 23;10(3):e0121862. doi: 10.1371/journal.pone.0121862 (PMC4370400; doi:10.1371/journal.pone.0121862)
Supplement: S4 Table — (DOC) [file pone.0121862.s004.doc]

S4 Table. Soil organic carbon stocks of black locust forests in semi-humid zone (Yongshou county).

| Forest ages (year) | Carbon stocks of each soil layer (Mg C ha^-1^) | | | | |
| --- | --- | --- | --- | --- | --- |
|  | 0－10 cm | 10－20 cm | 20－30 cm | 30－50 cm | 50－100 cm |
| 5 | 14.80 | 9.05 | 8.06 | 14.92 | 32.22 |
| 10 | 8.83 | 4.73 | 4.34 | 7.78 | 15.62 |
| 20 | 10.04 | 7.25 | 4.92 | 10.78 | 25.99 |
| 30 | 17.68 | 7.72 | 6.30 | 11.45 | 23.38 |
| 44 | 21.20 | 8.48 | 8.60 | 10.37 | 19.78 |
| 55 | 20.77 | 9.16 | 7.39 | 12.28 | 24.29 |
